# An Unusual Inverted Talar Neck Fracture–Dislocation

**DOI:** 10.1155/2022/8014529

**Published:** 2022-12-05

**Authors:** Brent Kokubun, Motasem Refaat

**Affiliations:** University of California at San Francisco Fresno, Fresno, CA, USA

## Abstract

Talar neck fractures occur on a continuum of injury severity. Hawkins classification, later modified by Canale, is the gold standard method of describing talar neck fractures by the degree of dislocation. It has proven to be clinically relevant in predicting risk of osteonecrosis. Despite its merits, talar neck fractures present on a wide spectrum of involvement of the body and neck, dislocation, and concomitant injuries, making every situation a challenge in treatment. We present a unique case of a talar neck fracture in which the talar dome had dislocated and inverted 180°, which is not described in the widely used Hawkins classification. We recommend urgent open reduction, low threshold for use of a transcalcaneal traction pin and dual incisions, and guarded prognosis of osteonecrosis and posttraumatic arthritis.

## 1. Introduction

Talar neck fractures are rare injuries that comprise less than 1% of all fractures [1]. They are often secondary to a high energy mechanism and result in high morbidity. Complications include posttraumatic arthritis, osteonecrosis, malunion, nonunion, and infection [2–4].

Hawkins classification, later modified by Canale, is the gold standard method of describing talar neck fractures by the degree of dislocation [5, 6]. It has proven to be clinically relevant in predicting risk of osteonecrosis [7, 8]. Despite its merits, talar neck fractures present on a wide spectrum of involvement of the body and neck, dislocation, and concomitant injuries, making every situation a challenge in treatment.

We present a unique case of a talar neck fracture in which the talar dome had dislocated and inverted 180°. This has been described specifically only once in previous literature [9]. Other reports have described bilateral talar neck involvement, entrapment of the neurovascular and tendinous structures, concomitant talar, malleolar, and calcaneal fractures [10–15]. We offer an updated report to highlight treatment decision-making in timing, reduction, and fixation, along with patient outcomes.

## 2. Statement of Informed Consent

The patient was verbally consented for inclusion of case details and imaging for publication. No identifying patient information is included. The patient agreed to these terms.

## 3. Case Presentation

**Figure 1:**
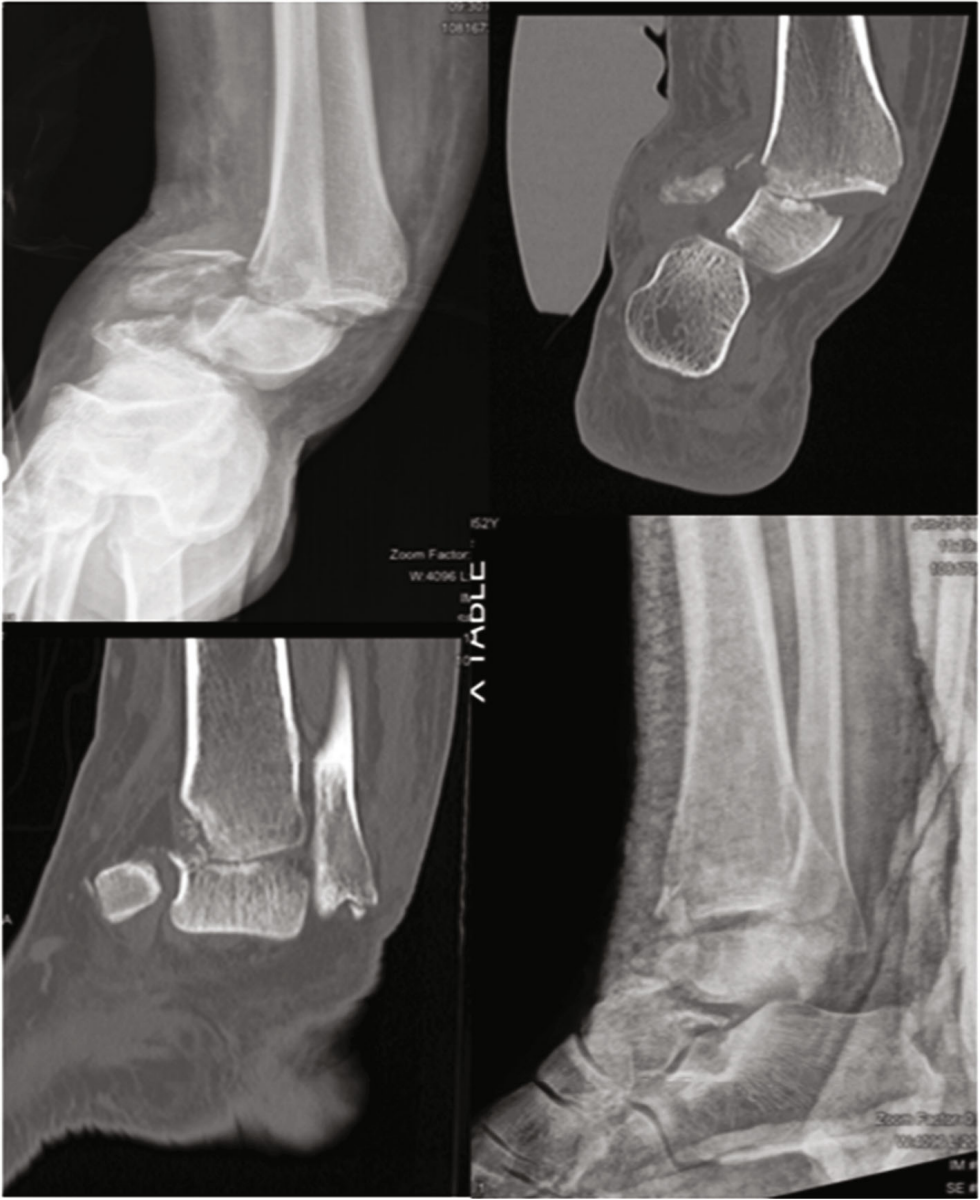
Injury films and CT scan demonstrated 180° inversion of proximal talus such that the dome was adjacent to the subtalar joint. The talus remained inverted after initial closed reduction attempt in the emergency department.

A 52-year-old female presented after a high speed motor vehicle collision with a chief complaint of right ankle pain. Physical exam noted a closed right ankle deformity displaced in valgus and internal rotation with medial skin bruising. She remained neurovascularly intact distally. Radiographs and computed tomography (CT) scan demonstrated a Hawkins 3 talar neck fracture–dislocation with a concomitant distal bimalleolar fracture. The proximal talar body was subluxed posteromedially, but rotated 180° such that the talar dome was adjacent to the subtalar joint (Figure 1). A single closed reduction attempt with splinting was performed in the emergency department to improve gross alignment of the ankle; however, the talus remained in an inverted position. The patient was cleared for urgent surgical intervention to prevent the risk of soft tissue compromise and was taken to the operating room (OR) within 5 hours of arrival to the emergency department.

**Figure 2:**
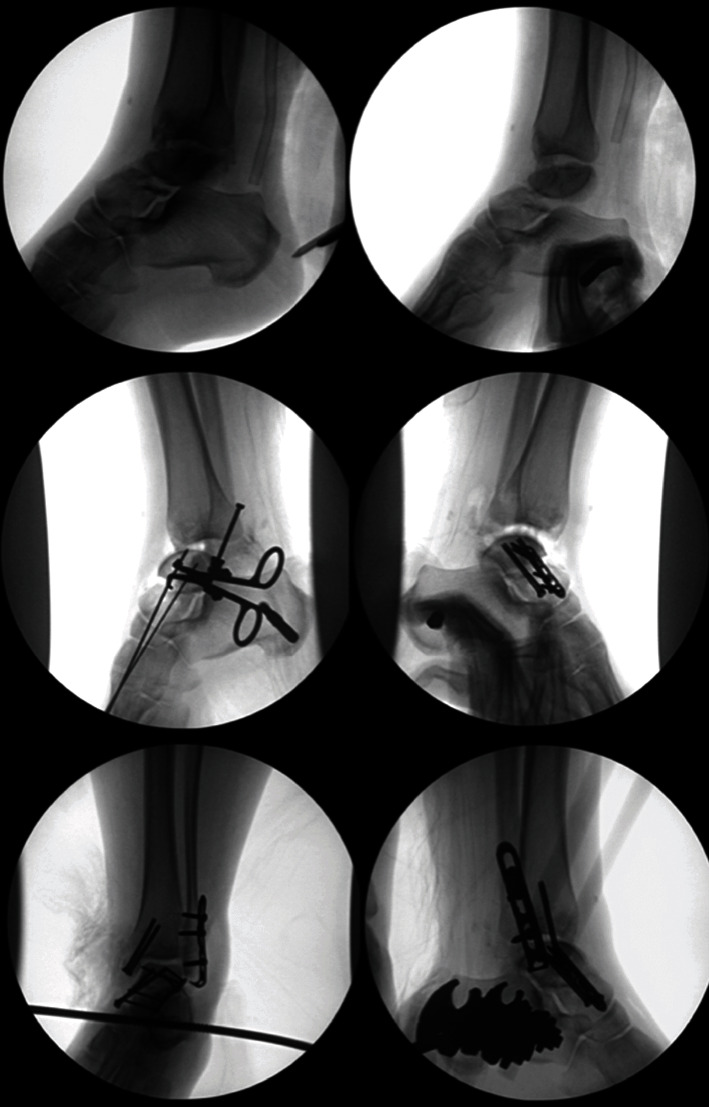
Intra-operative fluoroscopy demonstrates initial calcaneal pin traction, reduction through dual incision approach utilizing the bimalleolar fracture planes, mini fragment plate fixation of lateral talar neck and screw fixation of medial aspect, fixation of bimalleolar fracture, and final external fixator placement.

A transcalcaneal Shanz pin was first placed to apply axial traction to aid in reduction. Simultaneous anterolateral and anteromedial approaches to the talus were performed to visualize the inverted proximal talar segment. Working through the incisions and bimalleolar fracture planes, the proximal segment was manually rotated, so that the tibiotalar and subtalar joints were grossly reduced. The talar neck was reduced using K-wires and dental picks as joysticks. Definitive fixation was secured with a six-hole 2.0 mm bridge plate laterally and two cannulated cortical lag screws medially. Finally, the bimalleolar fracture was addressed appropriately, and an external fixator was applied to retain the alignment of the tibiotalar and subtalar joints (Figure 2).

**Figure 3:**
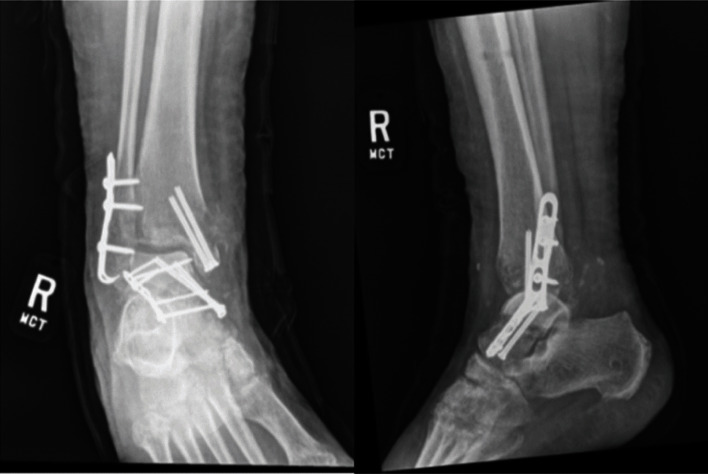
7-week follow-up; radiographs demonstrated sclerosis of talar dome and body with mild lucency of lateral dome suggestive of potential Hawkins sign.

**Figure 4:**
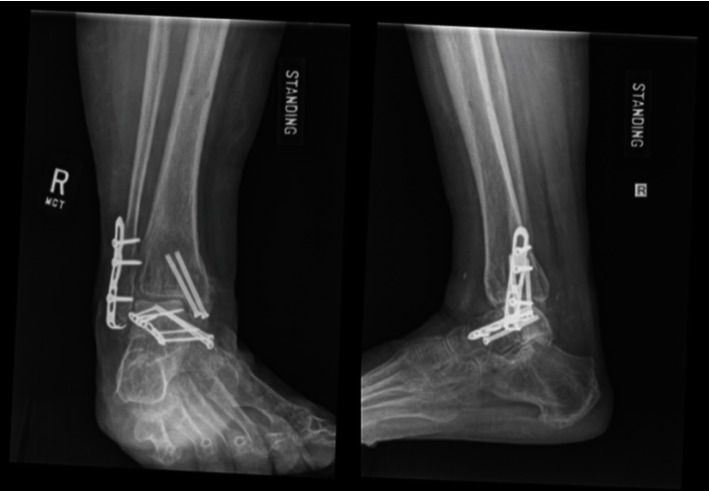
1-year follow-up; radiographs demonstrated union of fractures, early subtalar and tibiotalar arthritis, and improvement of prior sclerosis of talus without signs of collapse of talar dome.

Postoperatively the patient remained non weight-bearing for a total of 3 months. Her external fixator was removed in the OR 1 month after her index procedure without complications. At 7 weeks and 4 months postoperatively, radiographs revealed progressive sclerosis of the talar dome and body with possible lucency of the lateral dome (Figure 3). Her main complaint was ankle instability rather than pain as she was beginning to ambulate. She was referred to a foot and ankle orthopaedic specialist for discussion of fusion. At 1-year follow-up, radiographs demonstrated improvement of the sclerosis and no collapse of the talar dome (Figure 4). Her fractures had healed adequately with early signs of subtalar and tibiotalar arthritis. She deferred fusion and ambulates without significant pain or instability.

## 4. Discussion

The presented case demonstrates an unprecedented talar neck fracture pattern that remained a closed injury despite the significant dislocation. We found only one case report by Pantazopoulos et al. in 1972 that cited specifically 180° inversion of the talus [9]. Furthermore, we propose that the soft tissue stripping necessary to fully invert the proximal talus suggests a higher degree of damage not fully described by the Hawkins classification. This case may highlight an additional continuum of the injury pattern between a Hawkins 4 talar neck fracture–dislocation and open extrusion of the proximal talus. We recommend the surgeon determines the severity of the injury on a spectrum, rather than a rigid classification.

As part of a high volume Level 1 trauma center, we were equipped to treat our case in an efficient manner. Although timing of fixation has not been shown to be significant [2, 3, 16], the degree of dislocation indicated urgent reduction to preserve the soft tissue envelope. With interdisciplinary coordination, advanced imaging and operative intervention were achieved within 5 hours of arrival.

Open reduction was elected for immediately using a transcalcaneal Schanz pin and dual incisions. A dual incision approach working through her bimalleolar fracture provided adequate exposure that ultimately better preserved soft tissue structures and blood supply, such as the deltoid ligament [17, 18]. Simpson and Auston in 2016 detailed a similar technique and recommended calcaneal pin axial distraction, along with plantar flexion and rotation to maneuver the talar body so that the lateral process is delivered without abutting the lateral malleolus [19]. Other reports have supported use of a distractor, medial malleolar osteotomy, K-wire and/or Schanz pins, and arthroscopic-assisted reduction of the joint [20–24].

Although follow-up was 1 year, the time course was sufficient to demonstrate the patient's early outcome. Within 7 weeks postoperatively, radiographs demonstrated early sclerosis of the talar dome. At 4 months, adequate union of the fracture was achieved, but the sclerosis had progressed significantly. This complication aligns with previous literature that the degree of initial displacement and fracture comminution are significant predictors of osteonecrosis [7, 8].

Fortunately, at 1 year, our patient's radiographs demonstrated improvement of her sclerosis without signs of collapse, along with mild subtalar and tibiotalar arthritis. She deferred fusion and ambulates without significant pain. Evidence supports that these patients can have good outcomes with adequate open reduction internal fixation [25]. Although osteonecrosis has been reported at 49% after these injuries [2], Stone et al. in 2018 reported a high survivorship of talar neck fractures treated with open reduction internal fixation (ORIF). Only 1.9% of 1500 talar neck fractures required fusion within 4 years [26]. Anecdotally, at our institution, prior cases of significant talar neck displacement have demonstrated similar good clinical outcomes if expedited anatomic reduction is achieved.

In conclusion, this case demonstrates an unprecedented closed Hawkins 3 talar neck fracture–dislocation with 180° of rotational displacement. We recommend urgent open reduction, low threshold for use of a transcalcaneal traction pin and dual incisions for open reduction, and counseling the patient on guarded prognosis of osteonecrosis and posttraumatic arthritis.
